# Efficient backbone cyclization of linear peptides by a recombinant asparaginyl endopeptidase

**DOI:** 10.1038/ncomms10199

**Published:** 2015-12-18

**Authors:** Karen S. Harris, Thomas Durek, Quentin Kaas, Aaron G. Poth, Edward K. Gilding, Brendon F. Conlan, Ivana Saska, Norelle L. Daly, Nicole L. van der Weerden, David J. Craik, Marilyn A. Anderson

**Affiliations:** 1 Department of Biochemistry and Genetics, La Trobe Institute for Molecular Science, La Trobe University, Melbourne, Victoria 3086, Australia; 2 Division of Chemistry and Structural Biology, Institute for Molecular Bioscience, The University of Queensland, Brisbane, Queensland 4072, Australia

## Abstract

Cyclotides are diverse plant backbone cyclized peptides that have attracted interest as pharmaceutical scaffolds, but fundamentals of their biosynthetic origin remain elusive. Backbone cyclization is a key enzyme-mediated step of cyclotide biosynthesis and confers a measure of stability on the resultant cyclotide. Furthermore, cyclization would be desirable for engineered peptides. Here we report the identification of four asparaginyl endopeptidases (AEPs), proteases implicated in cyclization, from the cyclotide-producing plant *Oldenlandia affinis.* We recombinantly express *Oa*AEP1_b_ and find it functions preferably as a cyclase by coupling C-terminal cleavage of propeptide substrates with backbone cyclization. Interestingly, *Oa*AEP1_b_ cannot cleave at the N-terminal site of *O. affinis* cyclotide precursors, implicating additional proteases in cyclotide biosynthesis. Finally, we demonstrate the broad utility of this enzyme by cyclization of peptides unrelated to cyclotides. We propose that recombinant *Oa*AEP1_b_ is a powerful tool for use in peptide engineering applications where increased stability of peptide products is desired.

## Abstract (web summary)

Cyclotides are plant backbone-cyclised peptides with potential as pharmaceutical scaffolds. Here the authors report on the efficient backbone cyclization of cyclotides and unrelated peptides by a newly identified asparaginyl endopeptidase from *Oldenlandia affinis*.

## Graphical abstract

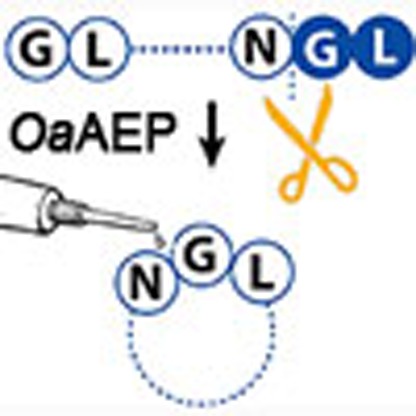

Proteases are abundant throughout nature and are essential for a wide range of cellular processes. They typically serve to hydrolyse polypeptide chains, resulting in either degradation of the target sequence or maturation to a biologically active form. Less frequently, proteases can also ligate polypeptides, producing new or alternatively spliced variants. This unusual function has been reported for processes such as the maturation of the lectin concanavalin A^1^, peptide presentation by major histocompatibility complex class I molecules^2^, and anchoring of bacterial proteins to the cell wall^3^. Recently, this enzymatic transpeptidation has also been implicated in the backbone cyclization of ribosomally synthesized cyclic peptides^4^,^5^,^6^,^7^,^8^,^9^,^10^.

Cyclotides are a well-studied class of gene-encoded cyclic peptides that are expressed in plants and exhibit a range of bioactivities including insecticidal, nematicidal and molluscicidal activity against agricultural pests^11^,^12^,^13^,^14^. Structurally, they are characterized by a cyclic cystine knot motif that confers exceptional stability. Importantly, this stable framework can be used as a pharmaceutical scaffold, and bioactive sequences have been successfully grafted into cyclotides^15^. Backbone cyclization can also endow peptides with oral bioavailability, suggesting that this modification might find broad application in peptide drug engineering^16^,^17^,^18^. However, *in vitro* cyclization of synthetic peptides is challenging and the limited availability of enzymes capable of this process is a hurdle to large-scale production^19^,^20^. Furthermore, expression yields of cyclotides in transgenic plants that are not native cyclotide producers is poor, impeding transfer of agriculturally relevant bioactivities to other plants^8^,^21^. The mechanism of enzymatic cyclization intrinsic to cyclotide biosynthesis is poorly understood. Elucidating it will be important for the realization of the pharmaceutical and agricultural potential of cyclotides and for increasing the cyclization efficiency of unrelated ‘designed' bioactive peptides.

Cyclotides are produced as precursors in which the cyclotide sequence is flanked by N- and C-terminal propeptides (Fig. 1). It is thought that enzymatic removal of the N-terminal propeptide precedes the final maturation step of C-terminal propeptide cleavage and ligation of the free N- and C-termini^8^,^21^. Only four native cyclases have been identified to date and the best characterized of these is the serine protease PatG, which cyclizes the bacterial cyanobactins^4^,^5^,^6^,^7^. In plants, the serine protease PCY1 cyclizes the segetalins; cyclic peptides from the Caryophyllaceae^4^. However, in the two other classes of plant-derived cyclic peptides (cyclotides and the PawS-derived cyclic peptides), strong Asx sequence conservation at the C-terminal P1 site implicates as possible cyclases the asparaginyl endopeptidases (AEPs), a group of cysteine proteases also known as vacuolar processing enzymes or Legumains, and this hypothesis is supported by studies in transgenic plants^8^,^9^,^21^,^22^.

Recently, an AEP (butelase 1) was isolated from the cyclotide-producing plant *Clitoria ternatea* and shown to cyclize a modified precursor of the prototypical cyclotide, kalata B1 (kB1) from *Oldenlandia affinis*, however, recombinant expression of functionally active butelase 1 has not been achieved, limiting its application^5^. Only one AEP with any cyclizing ability has been produced recombinantly, and this enzyme was highly inefficient, producing mainly hydrolysed substrate^10^. Here we report the identification, recombinant production and characterization of an *O. affinis* AEP that preferentially functions as a cyclase. The enzyme can cyclize native kalata substrate precursors and the unrelated anti-malarial peptide, R1, at close to 100% efficiency. This AEP releases the C-terminal propeptide of kB1, but it does not mediate the N-terminal processing event, which must occur first if efficient cyclization is to take place. Moreover, its specificity for model peptides mirrors the sequence requirements for cyclization of kB1 in transgenic plants, supporting a native function in the maturation of *O. affinis* cyclotides^8^,^21^.

## Results

### Identification and recombinant expression of *O. affinis* AEPs

**Figure 2:**
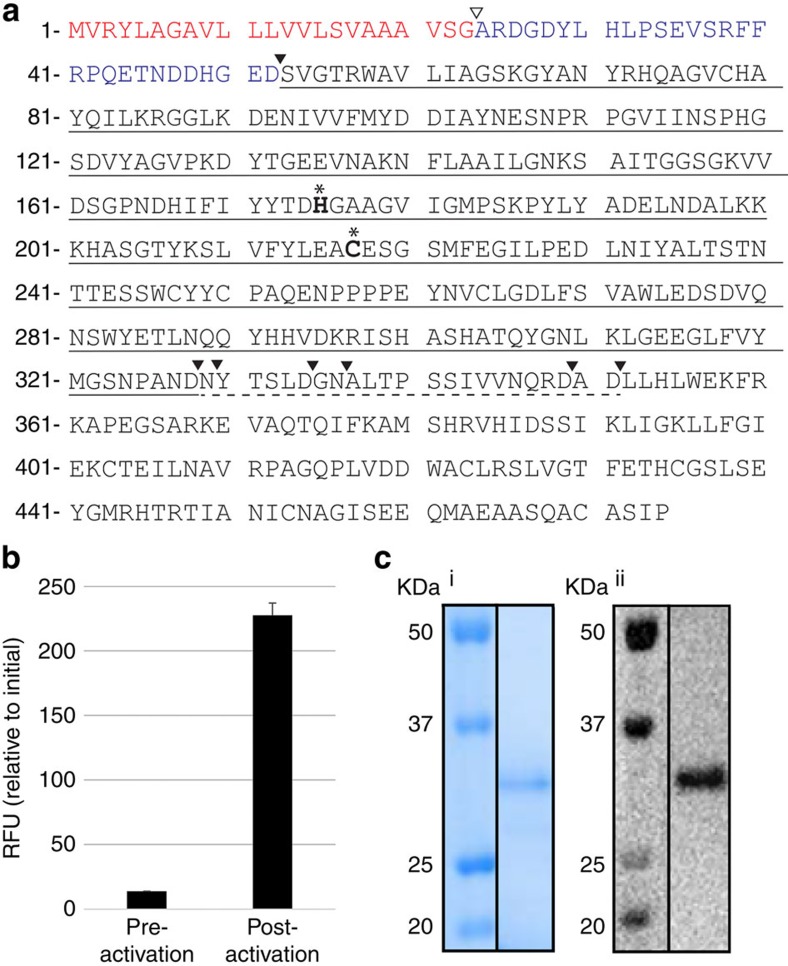
Expression of active *rOaAEP1*_*b*_ in *E. coli*. (**a**) Sequence of *Oa*AEP1_b_ predicted from *O. affinis* genomic DNA. Predicted ER signal sequence shown in red; N-terminal propeptide shown in blue; the putative signal peptidase cleavage site is indicated by ∇ and autocatalytic processing sites by ▾. The mature *Oa*AEP1 cyclase domain is underlined and the C-terminal auto-processing region is indicated with broken underline. The putative catalytic dyad is shown in bold and labelled with *. (**b**) An r*Oa*AEP1-containing anion exchange fraction pre-and post-activation at pH 4.5 (5 h, 37 °C) was tested for activity against the wt IQF peptide (14 μM). Baseline fluorescence from a no substrate control has been subtracted and the relative fluorescence intensity (RFU) at *t*=90 min is reported. The average of two technical replicates is shown and error bars report the range (**c**) Activated r*Oa*AEP1_b_ purified by cation exchange was analysed by SDS–PAGE and (i) Instant blue staining or (ii) Western blotting with anti-*Oa*AEP1_b_ (residues D_47_–P_474_) polyclonal rabbit serum.

Three expressed AEP isoforms were identified in an *O. affinis* complementary DNA library (*Oa*AEP1-3) and a fourth sequence, with a single nucleotide change from *Oa*AEP1 (resulting in a Glu_371_Val variant), was identified from genomic DNA (*Oa*AEP1_b_) (Fig. 2a; Supplementary Fig. 1). The four isoforms share at least 77% identity at the protein level, as determined by pairwise protein alignments. When compared with butelase 1, 64–69% identity was observed, whereas identity with human legumain was 49–53%.

*Oa*AEP1_b_ was expressed in *Escherichia coli* as a His6-ubiquitin-AEP1_b_ fusion protein (Supplementary Fig. 2a). AEPs are usually produced as zymogens that are self-processed at low pH to their mature, active form^23^,^24^,^25^. Consistent with this processing, activity of r*Oa*AEP1_b_ against an internally quenched fluorescent (IQF) peptide representing the native C-terminal processing site in kB1 (Table 1; wildtype (wt)) was markedly increased following incubation at pH 4.5 (Fig. 2b). After purification, a dominant band of ∼32 kDa was evident by reducing SDS–polyacrylamide gel electrophoresis (PAGE) and confirmed to be r*Oa*AEP1_b_ by Western blotting (Fig. 2c; Supplementary Fig. 2b). The average total protein yield from two independent experiments was ∼1.8 mg l^−1^ after activation and purification, however batch to batch variation in purity was observed. Although glycosylation of some AEPs has been reported^26^, the production of an active form in *E. coli* confirms that this is not a requirement for activity of *O. affinis* AEP1_b_.

Mass spectrometry (MS)/MS sequencing of peptide fragments generated from tryptic, chymotryptic or endoGlu-C digestion of the activated enzyme identified several peptide fragments with non-canonical cleavage sites, suggesting that they may be derived from r*Oa*AEP1_b_ auto-processing events (Supplementary Fig. 2). This allowed Asp52 to be assigned as the likely N-terminal auto-processing site and Asp328, Asn329, Asp334, Asn336, Asp349 and/or Asp351 as potential C-terminal processing sites (Fig. 2a; Supplementary Fig. 2). No peptides downstream of Asp351 were identified, indicating that the activation was essentially complete and that the C-terminal domain (Leu352–Pro474) was removed during the post-activation purification step. The theoretical mass of the processed forms (30.4–32.8 kDa) is in good agreement with that determined by SDS–PAGE/Western blotting (Fig. 2c).

Consistent with cysteine proteases of this class, r*Oa*AEP1_b_ was inhibited by iodoacetamide (1 mM), but was not affected by E64 (250 μM) or pepstatin A (10 μM) (Supplementary Fig. 3). Ac-YVAD-CHO (500 μM), a caspase-1 inhibitor reported to also inhibit AEPs^27^, was a poor inhibitor of the recombinant enzyme suggesting that at least some P′ residues are important for active site targeting.

### Substrate specificity

The activity of r*Oa*AEP1_b_ against IQF peptides representing wt and mutant versions of the native kB1 C-terminal cleavage site was determined (Table 1; Supplementary Fig. 4a). Along with the strict P1 Asx specificity characteristic of AEPs^24^, r*Oa*AEP1_b_ exhibited strong P2′ selectivity since after Leu_31_Ala substitution within the IQF peptide barely any hydrolysis was observed. This observation is consistent with the lack of cyclic product generated when the corresponding mutation was introduced *in planta*^21^. Kinetic parameters (*V*_max_, *K*_m_ and *k*_cat_) are reported where applicable (Table 1). The turnover rates (*k*_cat_) reported here (∼0.06–1.6 min^−1^) are much slower than that reported for recombinant human legumain assayed against a small substrate (∼8 s^−1^) (ref. 28). This is not unexpected given that r*Oa*AEP1_b_ prefers to carry out cyclization, rather than the hydrolysis being measured here. Supporting the observed P2′ selectivity, r*Oa*AEP1_b_ was unable to cleave the generic AEP substrate Z-AAN-MCA (Supplementary Fig. 5).

The substrate specificity of r*Oa*AEP1_b_ was compared with that of recombinant human legumain (rhuLEG; Supplementary Fig. 4b)^29^. A stringent P1 Asx requirement was again observed; however, in contrast to r*Oa*AEP1_b_, rhuLEG cleaved the Leu_31_Ala substrate at a rate similar to the wt substrate, demonstrating that P2′ specificity is not a feature of all AEPs.

### Cyclization of kB1 precursors

**Figure 3:**
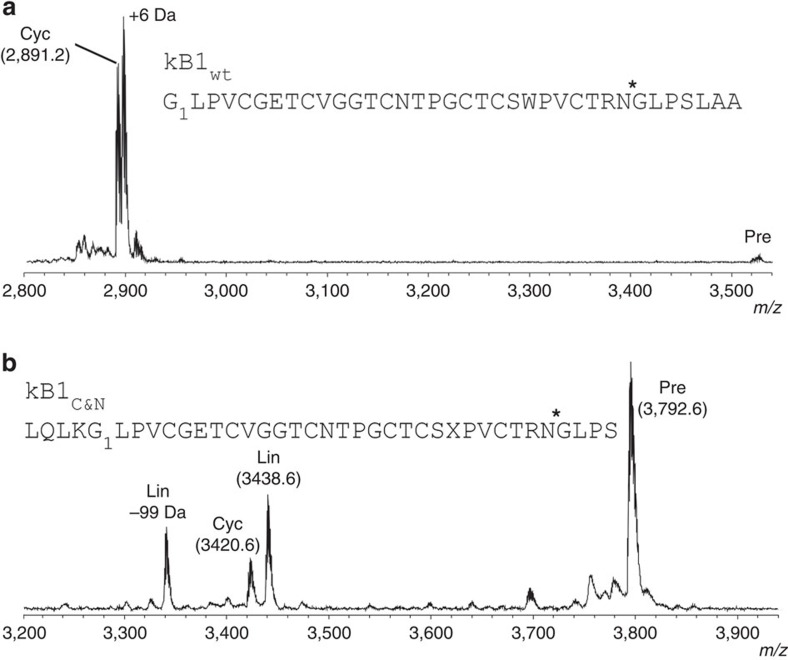
Enzymatic processing products of linear kB1 precursors. (**a**) MALDI MS profile of a kB1 precursor (kB1_wt_) containing the C-terminal propeptide in the presence of r*Oa*AEP1_b_ (22 h incubation). The +6 Da peak was observed only in the presence of reducing agent and corresponds to the reduced form of cyclic kB1. (**b**) MALDI MS profile of a kB1 precursor (kB1_C&N_) containing four C-terminal propeptide residues and four N-terminal propeptide residues in the presence of r*Oa*AEP1_b_ (20.5 h incubation). A side product originating from chemical synthesis likely represents a Val deletion (−99 Da). Data are representative of at least two technical replicates. X, benzoylphenylalanine. * denotes r*Oa*AEP1_b_ cleavage site. Observed monoisotopic masses (Da; [M+H]^+^) for dominant peaks are listed. Cyc, cyclic product; Lin, linear product; Pre, linear precursor.

To explore the cyclization ability of r*Oa*AEP1_b_, processing of correctly folded (as determined by NMR) synthetic kB1 precursors was assessed by MS. When incubated with the wt kB1 precursor carrying the native C-terminal pro-hepta-peptide (GLPSLAA), the active enzyme produced a peptide of 2,891.2 Da (monoisotopic, [M+H]^+^), consistent with the expected mass of mature, cyclic kB1 (Fig. 3a). This product was confirmed to be identical to native kB1 by reversed phase-high performance liquid chromatography (RP-HPLC) co-elution (Supplementary Fig. 6) and one- and two-dimensional-NMR experiments (Supplementary Fig. 7). Kinetic parameters (±s.e.m.) for the processing of the wt kB1 precursor were 0.53 (±0.1) s^-1^ for *k*_cat_, 212 (±76) μM for *K*_m_ and 2,500 M^−1^ s^−1^ for *k*_cat_/*K*_m_ as determined from a Michaelis–Menten plot (Supplementary Fig. 8). While the turnover rate (*k*_cat_) is lower than that reported for the plant-derived butelase 1 (17.08 s^−1^; ref. 5), it is far higher than that of the recombinantly expressed cyclase PatG (1 per day; ref. 7).

To determine if r*Oa*AEP1_b_ could also carry out the N-terminal processing required for cyclotide maturation, a kB1 precursor was tested that contained the folded cyclotide domain flanked by four residues from each of the N- and C-terminal propeptides (Fig. 3b). No N-terminal processing was observed, indicating that this processing is conducted by an enzyme other than *Oa*AEP1_b_. Although the bulk of the precursor remained intact after 20.5 h, the predominant processing product was a linear peptide lacking the C-terminal propeptide, demonstrating that correct N-terminal processing must occur before cyclization. Interestingly, a mass corresponding to a cyclized version of the C-terminally processed peptide (that is, C-terminal propeptide residues released, N-terminal propeptide residues remaining) was also observed, although this was the least abundant product.

### Processing of modified cyclotide precursors

**Figure 4:**
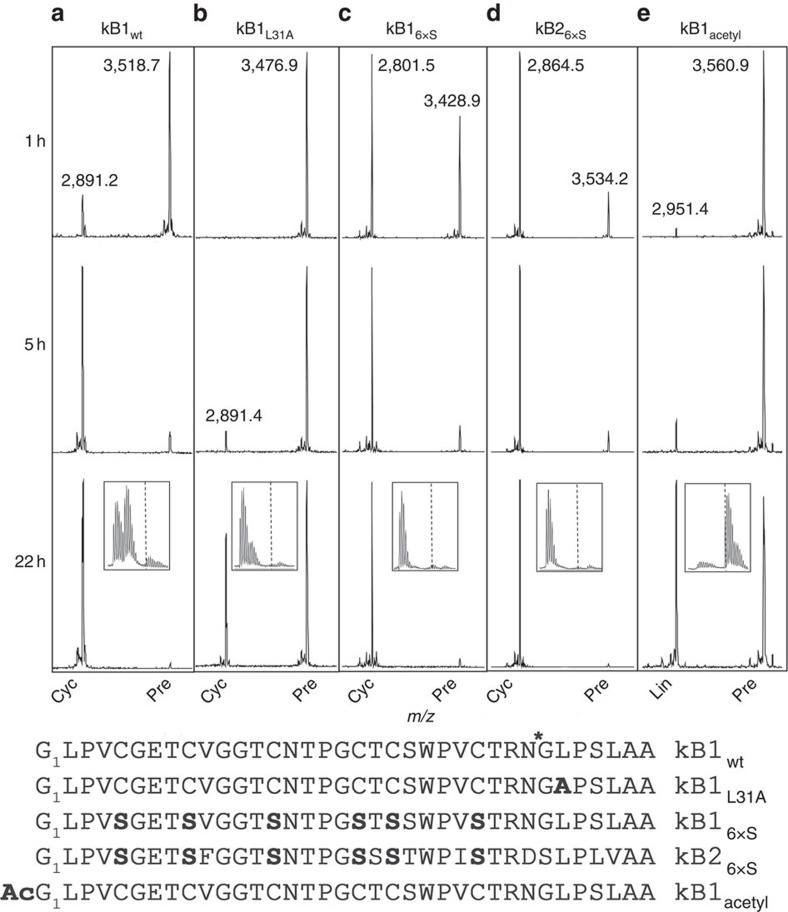
Modified linear kB1 and kB2 precursors are cyclized at different rates. MALDI MS spectra of (**a**) kB1_wt_, (**b**) kB1_L31A_, (**c**) kB1_6xS_, (**d**) kB2_6xS_ and (**e**) kB1_acetyl_ cyclotide precursors at 1, 5 and 22 h post-enzyme addition. Data are representative of three technical replicates. * denotes r*Oa*AEP1_b_ cleavage site. Observed monoisotopic masses (Da; [M+H]^+^) for dominant peaks are listed. Boxed inset at the 22 h time point zooms in on the region containing the processing product. Approximate positions of the monoisotopic mass of processed products is indicated by . Cyc, cyclic product; Lin, linear product; Pre, linear precursor.

To further probe cyclization requirements, we tested r*Oa*AEP1_b_-mediated processing of modified kB1 and kB2 precursors over time (Fig. 4). When presented within an IQF peptide, the Leu_31_Ala substrate analogue was not hydrolysed by r*Oa*AEP1_b_ (Table 1). However, the same substitution within the kB1 precursor did not preclude cyclization by r*Oa*AEP1_b_, although this version was cyclized far more slowly than the wt precursor (Fig. 4a,b). Surprisingly, the presence of disulfide bonds in the cyclotide precursors is not a requirement for cyclization since a kB1 substrate analogue in which all six cysteines were substituted with serines was also efficiently cyclized (Fig. 4c). The absence of a defined kB1-like structure in the kB1_6xS_ mutant was confirmed by NMR spectroscopy (Supplementary Fig. 7c). Similarly, a kB2 linear precursor with the same Cys→Ser substitutions was also efficiently cyclized, confirming that enzyme activity is not specific to individual cyclotides (Fig. 4d).

In cysteine protease-mediated peptide bond hydrolysis, nucleophilic attack of a water molecule is required to resolve the acyl-enzyme thioester intermediate. However, during peptide cyclization (or transpeptidation) the substrate's N-terminal amine is postulated to function as a competing nucleophile, facilitating aminolysis of the reactive thioester intermediate^30^. Accordingly, a kB1 precursor with an acetyl-capped N-terminal amine was processed only to a linear peptide lacking the C-terminal propeptide (Fig. 4e). This hydrolysis occurred at a slower rate than cyclization of the wt precursor (compare with Fig. 4a). Water can therefore access the active site of r*Oa*AEP1_b_, but cyclization is favoured over hydrolysis in the presence of an appropriately positioned nucleophile.

### Water is excluded during cyclization

**Figure 5:**
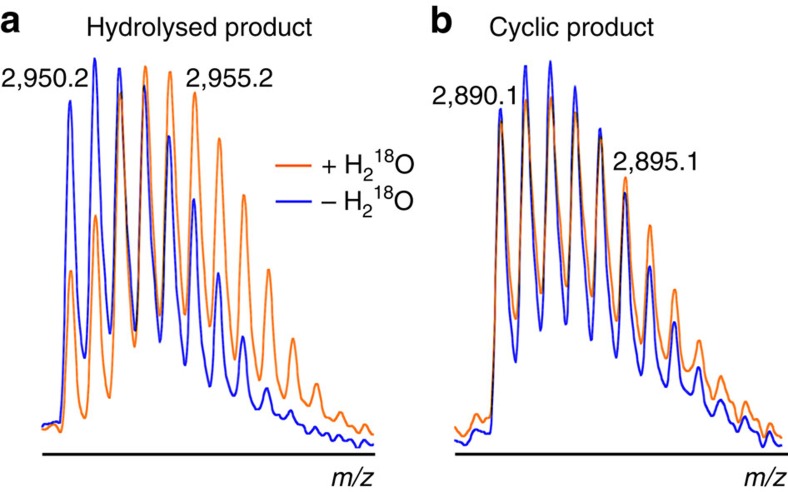
Enzymatic cyclization excludes water. MALDI MS profile of the enzymatic processing products of (**a**) kB1_acetyl_ and (**b**) kB1_wt_ linear precursors in the presence and absence of ^18^O-labelled water. An isotope shift indicative of ^18^O incorporation only occurs during hydrolysis. Observed masses of two isotopic peaks (Da; [M+H]^+^) are indicated. Data are representative of three technical replicates.

An alternative ligation mechanism, distinct from transpeptidation, was recently proposed for huLEG^31^). In that mechanism, initial hydrolysis of the C-terminal propeptide is followed by a separate ligation event requiring a C-terminal Asn residue in the substrate. To distinguish between these mechanisms in the case of r*Oa*AEP1_b_, reactions were carried out in the presence of ^18^O-labelled water and the products were analysed by high-resolution MS. An isotopic shift consistent with the incorporation of ^18^O was evident following enzymatic hydrolysis of the N-terminal acetylated kB1 precursor to give a linear product (Fig. 5a). However, there was no isotopic shift after processing of the wt precursor to a cyclic product, suggesting that hydrolysis is unlikely to play a role in cyclization by r*Oa*AEP1_b_ (Fig. 5a).

### rOaAEP1_b_ can cyclise an unrelated peptide

**Figure 6:**
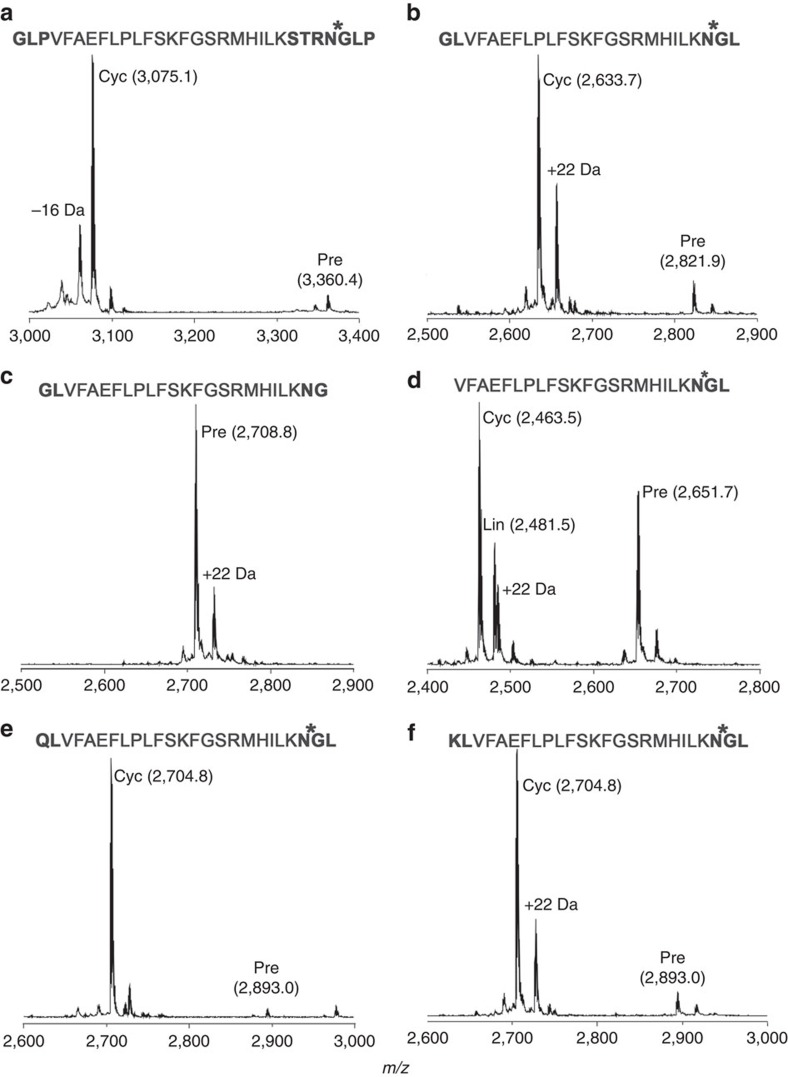
Flanking sequence requirements for cyclization of a model peptide by rOaAEP1. (**a**–**f**). MALDI MS spectra of the R1 peptide (VFAEFLPLFSKFGSRMHILK) with various flanking sequences 22 h post addition of r*Oa*AEP1_b_. Bold residues, flanking sequences. *denotes r*Oa*AEP1_b_ cleavage site. Observed monoiosotopic masses (Da; [M+H]^+^) are listed. +22 Da and −16 Da peaks present in some precursor and product spectra are likely to represent Na^+^ adducts and a synthesis-derived modification respectively. Data are representative of three technical replicates. Cyc, cyclic product; Lin, linear product; Pre, linear precursor.

We also investigated cyclization of other substrates structurally unrelated to cyclotides by r*Oa*AEP1_b_, focussing on the anti-malarial peptide R1 (refs 32, 33). This peptide was efficiently cyclized following the addition of N- and C-terminal AEP recognition sequences (Fig. 6a). Sequential trimming of the added recognition residues revealed that cyclization could be achieved following the addition of only a C-terminal Asn–Gly–Leu motif (although some linear product was also produced from this precursor) (Figs 6a–d). Lys and Gln were also accepted in place of Gly at the N terminus (Figs 6e–f) with little impact on yield at the time point tested. No processing of either the native R1 peptide or a modified R1 carrying the N-terminal Gly–Leu motif with only an Asn at the C terminus was observed (Supplementary Fig. 9). Subsequent digestion with endoGlu-C confirmed that, in all cases, r*Oa*AEP1_b_ processing produced cyclic peptide (Supplementary Fig. 10). Evidence of an additional, linear, r*Oa*AEP1_b_-generated cleavage product was only observed for the R1 variant without any N-terminal flanking residues (Fig. 6d; Supplementary Fig. 10c).

## Discussion

This study reports the cloning of four AEPs from the cyclotide-producing plant *O. affinis;* one of which was recombinantly expressed. The recombinant enzyme required self-processing to produce the active product: a cyclase that preferentially and efficiently couples C-terminal processing with C- and N-terminal ligation of linear *O. affinis* cyclotide precursors. Furthermore, this cyclizing ability was highly efficient when transferred to an unrelated anti-malarial peptide, demonstrating broad applicability in peptide engineering.

Consistent with other auto-inhibited proteases, r*Oa*AEP1_b_ required proteolytic activation to achieve maximum activity (Fig. 2b). The observed N-terminal auto-processing site (Asp52) is consistent with other experimentally validated N-terminal auto-processing sites identified in jack bean AEP^34^, butelase 1 (ref. 5) and human legumain^28^,^35^ (Fig. 2a, Supplementary Fig. 1). In contrast, six potential C-terminal auto-processing sites (Asp328/334/349/351, Asn329/336) were observed within a region particularly rich in Asn/Asp residues (324–351). This finding is in agreement with the multiple C-terminal maturation steps recently described for rhuLEG^28^,^35^. Regardless of which of these sites is relevant *in planta*, the instability of active AEPs above pH 6 (refs 28, 29, 36) will likely preclude direct production of active enzyme in *E. coli*. Activated r*Oa*AEP1_b_ proteolytically removed the C-terminal (but not N-terminal) propeptide of a kB1 precursor and resolved the acyl-intermediate in a hydrolysis-independent manner, generating a backbone cyclized product (Figs 3 and 5). r*Oa*AEP1_b_ could also hydrolyse precursors lacking a free N-terminal amine to produce linear products, albeit at a slower rate. Although it is unknown if *Oa*AEP1_b_ performs both these cyclase and protease activities *in vivo,* the observed preference for cyclization over hydrolysis suggests that it probably functions predominantly as a cyclase.

Dual protease/ligase capabilities have been reported for PatG^37^, a serine protease involved in cyclic peptide production in cyanobacteria, and more recently for human legumain^31^. Separate mechanistic pathways for hydrolysis and ligation have been proposed for human legumain: proteolysis proceeds via hydrolysis of a cysteinyl-thioester enzyme intermediate, whereas peptide ligation occurs via activation of the free peptidyl-α-carboxylate through transient formation of an enzyme-linked anhydride intermediate that is subsequently resolved via aminolysis^31^. The catalytically critical residue for this mechanism of ligation, Asp188, is conserved in *Oa*AEP1_b_, but several lines of evidence preclude a role for this pathway in *Oa*AEP1_b_-mediated peptide cyclization. First, r*Oa*AEP1_b_ cannot cyclize peptides carrying a free C-terminal Asn, the minimal proposed substrate requirement in the alternative pathway (Supplementary Fig. 9). Second, our H_2_^18^O experiments demonstrate the absence of ^18^O incorporation into the cyclized product, which strongly indicates that cyclization does not follow a hydrolysis/ligation mechanism as proposed in the alternative pathway (Fig. 5). Third, our MS/MS data for r*Oa*AEP1_b_ show no evidence of a reactive succinimide enzyme intermediate required for formation of the substrate–enzyme anhydrides^31^. Hence, our mechanistic data are in agreement with the traditional concerted mechanism, in which some of the energy from the (exergonic) cleavage of the C-terminal Asn-propeptide bond is preserved in the form of a thioester intermediate and used to overcome the energetically unfavourable (endergonic) peptide bond formation (cyclization) in the second step. However, alternative mechanisms may still play a role in cyclization mediated by other AEPs or with alternative substrates.

In the context of this established mechanism, C- and N-terminal proximity was thought to be crucial for cyclization to be favoured over hydrolysis^8^. Here we show that pre-organization of C- and N-termini in the substrate is not required by r*Oa*AEP1_b_ since unconstrained cyclotide precursors lacking the characteristic disulfide-bonded structure are efficiently cyclized (Fig. 4). Furthermore, r*Oa*AEP1_b_ can cyclize an anti-malarial peptide that is structurally and functionally unrelated to cyclotides following the addition of short flanking sequences (Fig. 6). These findings are consistent with the limited structural and/or sequence requirements imposed by other native cyclases on their substrates^4^,^5^,^7^ Conceivably, polypeptides of diverse composition and length may be cyclized by r*Oa*AEP1_b_, provided that association of the C- and N-termini is not sterically hindered.

The application of this technology is limited to peptides that can retain activity following incorporation of the additional residues required for AEP-mediated processing. While r*Oa*AEP1_b_ can cyclize a model peptide with only a single non-native residue incorporated to the mature peptide, this is at the cost of cyclization efficiency (Fig. 6d). Understanding the interplay between the sequence requirements for efficient cyclization and retention of bioactivity for a given target peptide will be crucial to realizing the potential of AEP-mediated cyclization. Importantly, the estimated turnover rate of r*Oa*AEP1_b_ (*k*_cat_, 0.53 s^−1^; Supplementary Fig. 8) is multiple orders of magnitude higher than the recombinantly produced cyclase PatG (1 d^−1^; ref. 7), supporting its widespread application in peptide engineering.

In a previous study, a conserved tripeptide motif C terminal to both the N- and C-terminal cyclotide processing sites was identified through cyclotide sequence analysis^8^. This motif is Gly–Leu–Pro in the kB1 sequence, and its importance for efficient cyclization is supported by mutagenesis studies in transgenic plants^8^,^21^. The protease specificity of r*Oa*AEP1_b_ reported here against IQF peptides mirrors these requirements (Table 1). At the C-terminal processing site, the P1 Asn and P2′ Leu are particularly well-conserved, and both were crucial for both *in planta* cyclization and *in vitro* cleavage of model peptides. However, over the longer incubation period of the cyclization assays, a kB1 precursor with a Leu_31_Ala mutation was still enzymatically processed to a cyclic product (Fig. 4). It was initially proposed that the conserved Leu residue at the P2′ position of cyclotides was important for preventing water from accessing the active site during cyclization. However the observed cyclization of the kB1 Leu_31_Ala mutant suggests that the role of a conserved bulky hydrophobic residue at the P2′ position is only to promote appropriate enzyme–substrate interaction. Congruent with this hypothesis, the absence of a P2′ residue renders substrates poor targets of both r*Oa*AEP1_b_ (cyclization and hydrolysis; Fig. 6c; Supplementary Fig. 5) and butelase 1 (at least for hydrolysis)^5^. In summary, our results suggest a cyclization model in which the cleaved C-terminal propeptide retains sufficient affinity to remain bound to the active site until it is displaced by the incoming N terminus of the peptide, finally leading to cyclization by resolving the acyl intermediate^8^,^38^.

This P2′ requirement is not characteristic of all AEPs^9^,^34^,^39^ and might be a predictor of cyclization ability within this protease family. Indeed, in extracts from *C. ternatea*, protein fractions that were active against the generic AEP substrate Z-AAN-MCA (which does not contain a P2′ residue) did not contain the cyclizing enzyme and, conversely, the butelase 1 containing fraction did not display activity against this substrate^5^. Here we report the presence of four unique AEP sequences in *O. affinis* (Fig. 2a; Supplementary Fig. 1), and demonstrate that one (*Oa*AEP1_b_) is capable of cyclizing *O. affinis* cyclotide precursors. Further work will investigate whether all, or a subset of, *O. affinis* AEPs (at least two more of which should exist to explain all the AEP contig sequences observed) exhibit this function and whether their substrate specificity is an accurate predictor of cyclization ability.

The constraints on the sequence of the incoming N terminus may not be very stringent. At least two residues with different properties are accepted in place of Gly at the P1′′ position by r*Oa*AEP1_b_ with comparable yields of cyclic product under the conditions tested (Fig. 6e,f). Butelase 1 and Pat G also exhibit promiscuity in this region^5^,^37^. Interestingly, Gly_1_ is highly conserved across cyclotides from different plant species, raising the possibility that selection at this position is not driven by AEP cyclase specificity. In transgenic plants, more stringent requisites were observed and no cyclic product was made from kB1 precursors with a conservative Gly_1_Ala mutation^8^. Because AEP cannot liberate the N-terminal propeptide, we hypothesize that selection at Gly_1_ might be driven by the putative N-terminal processing enzyme. Further analysis will be necessary to determine if this reflects differences in the enzyme homologues being assayed (that is, AEPs from the model plant *Nicotiana benthamiana* compared with AEPs from native cyclotide producers) or experimental conditions.

In conclusion, this study unequivocally demonstrates the involvement of an AEP in maturation of native *O. affinis* cyclotides, advancing our understanding of the biosynthesis of this important class of cyclic peptides. Furthermore, the promiscuous yet highly efficient nature of this recombinantly produced enzyme highlights its exciting potential value as a biological tool for cyclization of a range of bioactive peptides.

## Methods

### Peptide substrates and inhibitors

IQF peptides containing an N-terminal *o*-aminobenzoic acid (Abz) group and a C-terminal 3-nitrotyrosine (Y[3NO_2_]) were synthesized by Genscript at >90% purity. Control IQF peptides representing the predicted cleavage products of the wt peptide (Abz-STRN; GLPS-Y(3NO_2_) were also synthesized by Genscript at >90% purity. All IQF peptides were solubilized in 25% (v/v) acetonitrile:water. The fluorogenic peptide substrate Z-AAN-MCA (where Z is carboxybenzyl; MCA is 7-amido-4-methylcoumarin) and the caspase inhibitor Ac-YVAD-CHO (where Ac, acetyl; CHO, aldehyde) were supplied by the Peptide Institute and solubilized in dimethyl sulfoxide. The linear cyclotide precursor peptides kB1_wt_, kB1_C&N_, kB1_L31A_ and kB1_acetyl_ were chemically synthesized in-house by standard Fmoc solid-phase peptide synthesis. Folding and disulfide formation was carried out by incubating the reduced peptides in folding buffer (100 mM ammonium bicarbonate, 50% isopropanol, 2 mM reduced glutathione, 1 mM oxidized glutathione, pH 8.2) for 3 days^40^. The products were isolated by RP-HPLC at >95% purity and characterized by high-resolution MS and NMR spectroscopy. Peptides kB1_6xS_ and kB2_6xS_ as well as R1 and its derivatives were supplied by Genscript at >85% purity, as determined by RP-HPLC and MS. Peptides were dissolved in ultrapure water before analysis.

### *O. affinis* transcriptome

Total RNA was extracted from *O. affinis* root, leaf and seedling tissues using a phenol extraction method. Plant material was frozen in liquid nitrogen and ground to a fine powder, which was then resuspended in buffer (0.1 M Tris-HCl pH 8.0, 5 mM EDTA, 0.1 M NaCl, 0.5% SDS, 1% 2-mercaptoethanol), extracted twice with 1:1 phenol:chloroform and precipitated by addition of isopropanol. The pellets were dissolved in 0.5 ml water and RNA was precipitated overnight at 4 °C by addition of 4 M lithium chloride. The extracted RNA of each tissue was analysed by GeneWorks using the Illumina GAIIx platform. In total, 69.3 million 75 bp paired-end reads were generated. Reads were filtered with a phred confidence value of Q37 and assembled into contigs using Oases^41^ with k-mer ranging from 41–67. The assemblies were merged using cd-hit-est^42^, resulting in 270,000 contigs. Statistics on the depth of sequencing were made by aligning the reads of each tissue on the contigs using BWA^43^. All the sequences, including one AEP, previously identified from an EST library of *O. affinis* were present among the contigs^44^. Homologues of this AEP sequence were searched using BLAST^45^ in the contig library using a maximum E-value of 1e-20, resulting in the identification of 371 putative AEP transcripts. These sequences could then be clustered in 13 groups sharing at least 90% sequence identity using cd-hit^42^.

### OaAEP1-3 cloning

Full-length AEP transcripts from the *O. affinis* transcriptome assembly were used to design a set of primers. A single degenerate forward primer (*Oa*AEPdegen-F, 5′-ATG GTT CGA TAT CYC GCC GG-3′) was sufficient to amplify all sequences since variability within the extreme 5′ region of each full-length transcript was limited to a single nucleotide position. Three reverse primers (*Oa*AEP1-R, 5′-TCA TGA ACT AAA TCC TCC ATG GAA AGA GC-3′; *Oa*AEP2-R, 5′-TTA TGC ACT GAA TCC TTT ATG GAG GG-3′; *Oa*AEP3-R 5′-TTA TGC ACT GAA TCC TCC ATC G-3′) were designed with the aid of Primer 3 (ref. 46). Each primer set successfully amplified an AEP sequence.

To clone expressed *Oa*AEPs, total RNA was extracted from *O. affinis* leaves and shoots using TRIzol (Life Technologies) and was reverse transcribed with SuperScript III reverse transcriptase (Life Technologies) according to the manufacturer's instructions^11^. Target sequences were amplified from the resulting complementary DNA using Phusion High Fidelity Polymerase (New England BioLabs) and the primers described above under the recommended reaction conditions. Gel extracted PCR products were dA-tailed by incubation with Invitrogen Taq Polymerase (Life Technologies) and 0.5 μl 10 mM dA in the supplied buffer. The processed products were cloned into pCR8-TOPO (Life Technologies) and transformed into *E. coli*. Purified DNA from clones that were PCR positive for an AEP insert were sent for Sanger sequencing at the Australian Genome Research Facility (www.agrf.org). Coding sequences have been deposited in Genbank (accession codes: *Oa*AEP1 (KR259377), *Oa*AEP2 (KR259378), *Oa*AEP3 (KR259379)).

In an alternative approach, genomic DNA was extracted from *O. affinis* leaf tissue using a DNeasy Plant Mini Kit according to the manufacturer's instructions. PCR amplification from this DNA used primers specifically targeting the *Oa*AEP1 nucleotide sequence. Gel extracted product was dA-tailed as above, cloned into the TOPO vector and transformed into *E. coli.* Sequencing of PCR-positive clones identified a fourth sequence with a single amino acid change from *Oa*AEP1 (*Oa*AEP1_b_).

### Antibodies

Polyclonal anti-*Oa*AEP1_b_ rabbit serum was generated by immunizing a New Zealand White rabbit with a denatured, inactive form of *Oa*AEP1_b_ (residues D_47_–P_474_) that was produced recombinantly in *E. coli*. The rabbit received three doses, four weeks apart, of 150 μg of antigen in 50% (v/v) PBS and Freund's incomplete adjuvant. Serum was obtained 2 weeks after the final dose and used at a 1:2,000 dilution for Western blotting.

### Recombinant expression of *O. affinis* AEP1_b_ (r*Oa*AEP1_b_)

Initial trials to produce active *O. affinis* AEP1_b_ based on predicted N- and C-terminal processing sites (residues D_47_–D_420_) were unsuccessful and subsequent expression attempts incorporated both N- and C-terminal prodomains. DNA encoding full-length *O. affinis* AEP1_b_ without the putative signalling domain (residues A_24_-P_474_) was inserted into the pHUE vector^47^ to give a His6-ubiquitin-*Oa*AEP1_b_ fusion protein construct (Supplementary Fig. 2) and introduced into T7 shuffle *E. coli* cells (New England BioLabs). Transformed cells were grown at 30 °C in superbroth (3.5% tryptone (w/v), 2% yeast extract (w/v), 1% glucose (w/v), 90 mM NaCl, 5 mM NaOH) to mid-log phase; the temperature was then reduced to 16 °C and expression was induced with isopropyl ß–D-1-thiogalactopyranoside (0.4 mM) for ∼20 h. Cells were harvested by centrifugation and resuspended in non-denaturing lysis buffer (50 mM Tris-HCl, 150 mM NaCl, 0.1% triton X 100, 1 mM EDTA, pH 7). Lysis was promoted by a total of five freeze/thaw cycles and the addition of lysozyme (hen egg white; 0.4 mg ml^−1^). DNase (bovine pancreas; 40 μg ml^−1^) and MgCl_2_ (0.4 M) were also added. Cellular debris was removed by centrifugation and the lysate was stored at −80 °C until required.

### Purification and activation of rOaAEP1_b_

Lysate containing expressed r*Oa*AEP1_b_ was filtered through a 0.1 μm glass fibre filter before being diluted 1:8 in buffer A (20 mM bis-Tris, 0.2 M NaCl, pH 7) and loaded onto two 5 ml HiTrap Q Sepharose high performance columns connected in series (GE Healthcare; 1.6–3.1 ml undiluted lysate per millilitre resin). Bound proteins were eluted with a continuous salt gradient (0–30% buffer B (20 mM bis-Tris, 2 M NaCl, pH 7); 15 column volumes) and AEP-positive fractions identified by Western blotting (anti-*Oa*AEP1_b_ rabbit serum (1:2,000); peroxidase-conjugated anti-rabbit IgG (GE Healthcare NA934; 1:5000)). To self-activate r*Oa*AEP1_b_, EDTA (1 mM) and Tris(2-carboxyethyl)phosphine hydrochloride (0.5 mM) were added, the pH was adjusted to 4.5 with glacial acetic acid and the protein pool was incubated for 5 h at 37 °C. Protein precipitation at this pH allowed removal of the bulk of the contaminating proteins by centrifugation. The remaining protein was filtered (0.22 μm), diluted 1:8 in buffer A2 (50 mM acetate, pH 4) then captured on a 1 ml HiTrap SP Sepharose high performance column (GE Healthcare). Bound proteins were eluted with a salt gradient (0–100% buffer B2 (50 mM acetate, 1 M NaCl, pH 4); 10 column volumes) and AEP-positive fractions were pooled. The final product was analysed by MS and reducing SDS–PAGE followed by Western blotting and staining with Instant blue (Expedeon). The total concentration of protein was estimated by bicinchoninic acid assay according to the manufacturer's instructions.

### Identification of the auto-processing sites of rOaAEP1_b_

Aliquots of r*Oa*AEP1_b_ (5 μl) were diluted 1:1 with either 100 mM ammonium bicarbonate pH 8.0 (trypsin, chymotrypsin) or 100 mM ammonium phosphate pH 8.0 (endoGlu-C) and enzymatically digested with endoGlu-C, trypsin or chymotrypsin (100 ng μl^−1^). Cleavages were conducted over 16 h at 37 °C (endoGlu-C, trypsin) or 30 °C (chymotrypsin). Injections of each digest (5 μl) were introduced to a Shimadzu nanoLC delivering a linear acetonitrile gradient at a flow rate of 500 nl min^−1^ for reversed-phase separation on a C18 Zorbax column (Agilent 300SB-C18, 3.5 μm particle size, 150 mm × 100 μm). Column eluate was interfaced directly with a 5600 TripleTOF LC-MS/MS instrument (AB SCIEX, Canada) equipped with a nanoelectrospray ionization source.

Tandem MS data were generated in Information Dependent Acquisition experiments, wherein full-scan TOF-MS spectra were acquired for 250 ms over *m/z* 350–1,800, and the 20 most intense signals with charge state +2 to +5 were selected for product ion scans of duration 50 ms over *m/z* 80–1,400. Data were acquired and processed using the Analyst TF 1.6 software (AB SCIEX, Canada). Automated protein identification was performed in ProteinPilot 4.0 using the Paragon algorithm with ‘no enzyme' settings to interrogate an *E. coli* K12 proteome database (Uniprot) concatenated with sequences of r*Oa*AEP1_b_ and those of the three cleavage enzymes, with false discovery rate set at *P*<0.05. Spectral data for peptides software matched to r*Oa*AEP1_b_ were confirmed via manual interpretation in Analyst TF 1.6.

### Assaying protease activity against fluorescent peptides

To assay activity of r*Oa*AEP1_b_ against both internally quenched and other fluorescent peptides, substrate and enzyme were diluted as appropriate in activity buffer (50 mM sodium acetate, 50 mM NaCl, 1 mM EDTA, 0.5 μM Tris(2-carboxyethyl)phosphine hydrochloride, pH 5). To assay activity of rhuLEG (R&D systems) against the same substrates, the enzyme was first activated by incubation in 50 mM sodium acetate, 100 mM NaCl, pH 4 (4 μl buffer/1 μl enzyme) for 2 h at 37 °C. Substrates and activated rhuLEG were diluted in 50 mM MES, 250 mM NaCl, pH 5 as required. Diluted enzyme and substrate were added to black, flat bottomed microtiter plates in a total assay volume of 110–200 μl. The change in fluorescence intensity over time was monitored on a SpectraMax M2 (Molecular Devices) using excitation/emission wavelengths of 320/420 nm (IQF peptides) or 360/460 nm (other fluorescent peptides). Substrate and enzyme concentrations in each assay and the time point presented are as indicated in the figure legends.

To determine the kinetics of r*Oa*AEP1_b_ activity against IQF peptides, each substrate was assayed at a range of concentrations between 2.5 and 80 μM in a total volume of 200 μl. The total protein concentration of the enzyme preparation used in the kinetic assays was 3.5 μg ml^−1^. It was not possible to precisely determine the concentration of active enzyme due to impurities remaining in the preparation and the absence of an inhibitor appropriate for active site titration. However, a conservative turnover rate (*k*_cat_) was estimated based on a mass of 32 kDa and the assumption that the total protein concentration reflected active enzyme. At each substrate concentration, initial velocities were calculated from the linear portion of the progress curve. *K*_m_ and *V*_max_ were estimated using the Michaelis–Menten equation and the curve-fitting program GraphPad Prism (GraphPad Software, San Diego).

The high peptide concentrations required for estimating kinetic parameters necessitated the use of a correction factor to account for the inner filter effect; a phenomenon where high relative concentrations of the quenching group impede detection of the signal from the fluorescent donor even after substrate hydrolysis^48^. This was achieved as described previously^48^. The output generated by the fluorescent hydrolysis product (Abz-STRN) was measured in the presence of each concentration of non-hydrolysed substrate. The correction factor was the ratio between the expected and observed fluorescence signal at each substrate concentration. The corrected signal for each data point was then converted to amount of product by comparison to a standard curve of the fluorescent hydrolysis product.

### Inhibition assays

To investigate the impact of inhibitors on enzyme activity against the wt IQF peptide, r*Oa*AEP1_b_ (4.4 μg ml^–1^ total protein) was incubated with the indicated concentration of E64, Ac-YVAD-CHO, pepstatin A and iodoacetamide for 40 min before addition to the substrate (11 μM). Enzyme activity against the wt IQF peptide was then assessed as described above.

### Cyclization assay

Linear target peptides (280 μM) were incubated with r*Oa*AEP1_b_ (12 μg ml^−1^ total protein unless otherwise indicated) in activity buffer. The reaction was allowed to proceed for up to 22 h at room temperature and was analysed by matrix-assisted laser desorption/ionization MS (MALDI MS), RP-HPLC or NMR as appropriate.

To confirm the presence of cyclic product, R1 derivatives processed by r*Oa*AEP1_b_ were subsequently digested with endoGlu-C (25 μg ml^−1^) in reaction buffer (50 mM Tris-HCl, 0.5 mM Glu–Glu, pH 8) such that the final dilution of the cyclization mix was 1:4. The reaction was allowed to proceed for 18 h at 37 °C before analysis by MALDI MS.

In heavy water experiments, isotopically labelled water (97 atom % ^18^O) was used in place of unlabelled water. Linear target peptides (70 μM) were incubated with r*Oa*AEP1_b_ (6 μg ml^−1^ total protein) in a non-reducing activity buffer (50 mM sodium acetate, 50 mM NaCl, 1 mM EDTA, pH 5) for 22 h at room temperature. The final H_2_^18^O concentration in the assay was 81%.

### MS to track cyclization of linear peptides

Cyclization of linear target peptides was monitored by MALDI MS. The reaction mixture (10–20 μl) was desalted using C18 zip tips and eluted in 4 μl 50% acetonitrile, 0.1% trifluoroacetic acid (TFA). A saturated MALDI matrix solution (α–cyano-4-hyroxycinnamic acid) prepared in 95% acetonitrile, 0.1% TFA was diluted 1:22 such that the final matrix solution comprised 90% acetonitrile, 0.1% TFA and 1 mM NH_4_H_2_PO_4_. Eluted samples were mixed 1:4 with the MALDI matrix, spotted onto a MALDI plate and analysed by an Ultraflex III TOF/TOF (Bruker) in positive reflector mode.

### Purification of kB1 following in vitro cyclization

The crude cyclization mixture was loaded to an Agilent Zorbax C18 reversed-phase column (4.6 × 250 mm, 300 Å) and separated on a Shimadzu Prominence system using a linear gradient of 5–55% buffer B (90% acetonitrile, 10% H_2_O, 0.05% TFA) in buffer A (0.05% TFA/H_2_O) over 60 min. Fractions were collected manually, analysed by MALDI MS essentially as described above and lyophilized. Analytical HPLC and co-elution studies with chemically synthesized kB1 were carried out as described above.

### Nuclear magnetic resonance spectroscopy

All peptides were dissolved in 90% H_2_O/10% D_2_O at a concentration of ∼2.0 mg ml^−1^ (0.5–0.75 mM). kB1 obtained from *in vitro* cyclizations was dissolved at 0.3 mg ml^−1^ (∼0.1 mM). Spectra were recorded on a Bruker Avance 600 MHz spectrometer equipped with a cryoprobe at 298 K. Phase-sensitive mode using time-proportional phase incrementation for quadrature detection in the *t*_1_ dimension was used for all two-dimensional spectra. Excitation sculpting with gradients was used to achieve water suppression. NMR experiments included TOCSY using a MLEV-17 spin lock sequence with an 80 ms mixing time, and NOESY with a 200 ms mixing time. Spectra were recorded with 4,096 data points in the *F*_2_ dimension and 512 increments in the *F*_1_ dimension. The *t*_1_ dimension was zero-filled to 1,024 real data points, and the *F*_1_ and *F*_2_ dimensions were multiplied by a sine-squared function before Fourier transformation. The spectra were referenced to the water signal at 4.77 p.p.m. at 298 K. All spectra were processed using TopSpin (Bruker) and manually assigned with CCPNMR using the sequential assignment protocol.

### Cyclization kinetics

To determine the kinetics of r*Oa*AEP1_b_ activity against the wt kB1 precursor, the substrate was assayed at room temperature at a range of concentrations between 75 and 250 μM in a total volume of 20–160 μl of activity buffer. The total protein concentration of the enzyme preparation added to the kinetic assays was 19.7 μg ml^−1^. The reaction was quenched after 5 min with 0.1% TFA and the volume adjusted to 800 μl. A volume of 700 μl was loaded onto a reversed-phase C18 analytical column (Agilent Eclipse C18, 5 μm, 4.6 × 150 mm) and peptides were separated by HPLC (19 min linear gradient of 12–60% acetonitrile, 0.1% TFA at 1 ml min^−1^). The identity of eluted peaks was confirmed using MALDI MS. The area under the curve corresponding to the precursor peptide was quantitated by comparison to a standard curve and initial velocities were calculated by converting this to μmoles product formed. Kinetic parameters were estimated using the Michaelis–Menten equation and the curve-fitting program GraphPad Prism (GraphPad Software, San Diego). As for the IQF peptides, it was not possible to precisely determine the concentration of active enzyme due to impurities remaining in the preparation and the absence of an inhibitor appropriate for active site titration. However, a conservative turnover rate (*k*_cat_) was estimated based on a mass of 32 kDa and the assumption that the total protein concentration reflected active enzyme. Differences in enzyme preparations means these parameters are not directly comparable to those determined for the IQF peptides.

## Additional information

**How to cite this article:** Harris, K. S. *et al.* Efficient backbone cyclization of linear peptides by a recombinant asparaginyl endopeptidase. *Nat. Commun.* 6:10199 doi: 10.1038/ncomms10199 (2015).

## Supplementary Material

Supplementary Information Supplementary Figures 1-10.

## Figures and Tables

**Figure 1:**
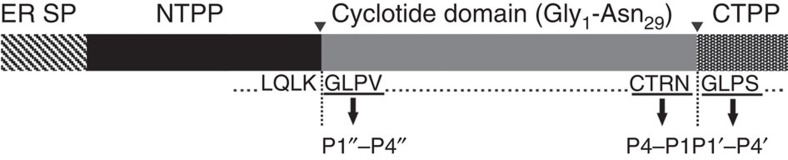
Schematic representation of the *Oak1* gene. The precursor protein encoded by the *Oak*1 gene is proteolytically processed to mature kB1. ▾ indicates the N- and C-terminal processing sites. r*Oa*AEP1_b_ targets the C-terminal processing site. The C-terminal P1/P1′ - P4/P4′ sites are indicated. P1′′-P4′′ denote the N-terminal residues that replace the P1′-P4′ residues on release of the C-terminal propeptide. CTPP, C-terminal propeptide; ER SP, endoplasmic reticulum signal peptide; NTPP, N-terminal propeptide.

**Table 1:** Kinetic parameters of IQF peptide cleavage by r*Oa*VPE1_b_.

**IQF peptide**	Sequence*	***V***_**max**_ **(nmoles min**^**-1**^ **mg**^**-1**^ **protein) (**±**s.e.m.)**†	***K***_**m**_ **(μM) (**±**s.e.m.)**†	***k***_**cat**_ **(min**^**-1**^**) (**±**s.e.m.)**†‡
wt	Abz-STRN↓GLPS-Y(3NO_2_)	51.3 (±5.8)	55.0 (±6.4)	1.6 (±0.2)
R_28_A	Abz-STAN↓GLPS-Y(3NO_2_)	6.9 (±0.6)	13.0 (±2.4)	0.2 (±0.02)
R_28_K	Abz-STKN↓GLPS-Y(3NO_2_)	29.3 (±3.5)	42.0 (±4.0)	0.9 (±0.1)
N_29_A	Abz-STRA↓GLPS-Y(3NO_2_)	NA§	−	−
N_29_Q	Abz-STRQ↓GLPS-Y(3NO_2_)	NA§	−	−
N_29_D	Abz-STRD↓GLPS-Y(3NO_2_)	∼2||	ND||	∼0.06||
G_30_A	Abz-STRN↓ALPS-Y(3NO_2_)	51.0 (±2.0)	29.0 (±1.4)	1.6 (±0.07)
G_30_S	Abz-STRN↓SLPS-Y(3NO_2_)	35.5 (±2.7)	31.4 (±2.5)	1.1 (±0.08)
L_31_A	Abz-STRN↓GAPS-Y(3NO_2_)	NA§	−	−
L_31_I	Abz-STRN↓GIPS-Y(3NO_2_)	ND¶	ND¶	ND¶

IQF, internally quenched fluorescent; NA, no activity; ND, not determined.

^*^IQF peptide residues are numbered according to their position within the native kB1 precursor, where the mature cyclotide incorporates Gly_1_-Asn_29_; native Cys_26_ is substituted with Ser to avoid unpaired Cys residues.

^†^*N*≥ 3;±standard error of the mean (s.e.m.)

^‡^*k*_cat_ is a conservative estimate assuming that the total concentration of active enzyme is equal to the total protein concentration in the enzyme preparation and an enzyme mass of 32 kDa.

^§^No activity detected under the conditions tested (up to 80 μM substrate; up to 6 h incubation).

^||^Low *V*_max_ precluded accurate estimation of kinetic parameters.

^¶^*K*_m_ above the range of the substrate concentrations used in this analysis precluded accurate estimation of kinetic parameters.
