# Supplementary material for: Efficient backbone cyclization of linear peptides by a recombinant asparaginyl endopeptidase
Source: Nat Commun. 2015 Dec 18;6:10199. doi: 10.1038/ncomms10199 (PMC4703859; doi:10.1038/ncomms10199)
Supplement: Supplementary Information — Supplementary Figures 1-10. [file ncomms10199-s1.pdf]

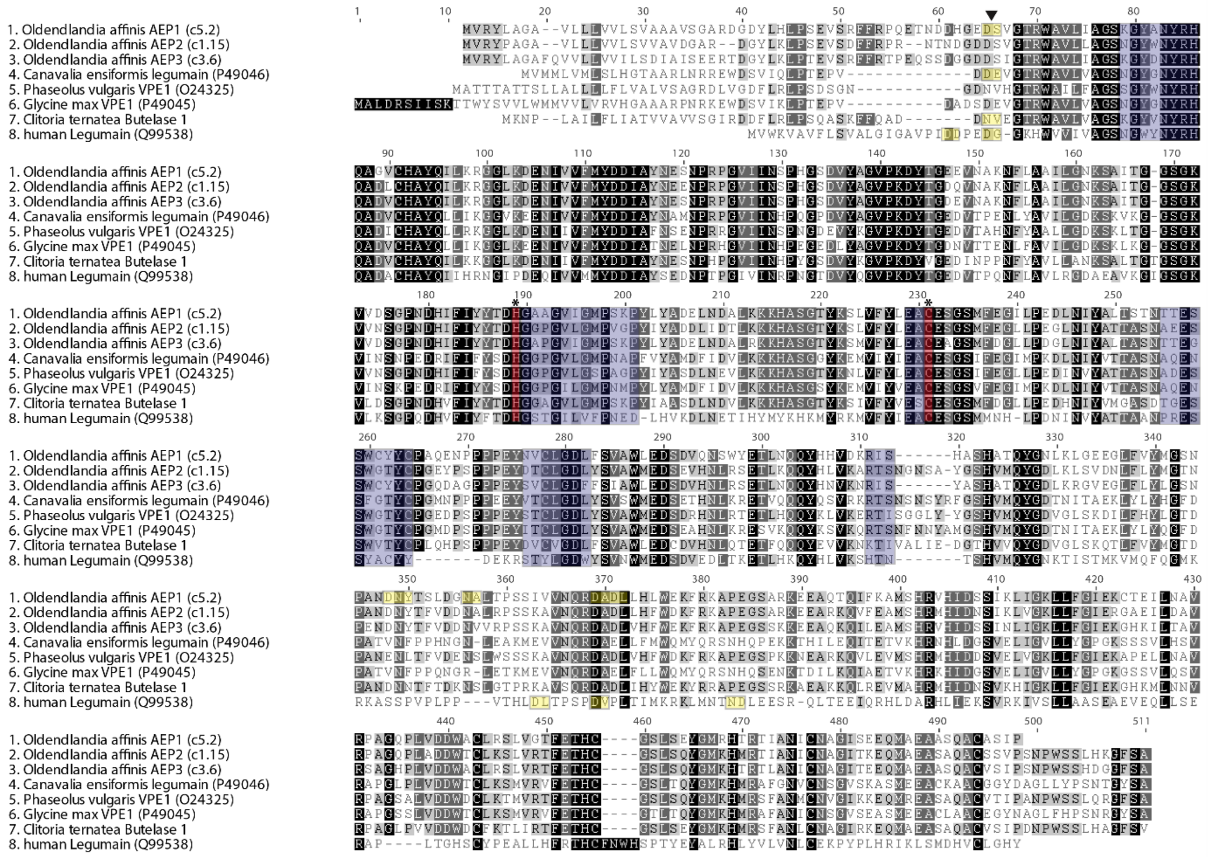


**Supplementary Figure 1:** *Sequence alignment of AEP homologues.* Experimentally validated auto-processing sites (N- and C-terminal) are highlighted in yellow. ▼ shows the N-terminal r*Oa*AEP1$_b$ processing site. Catalytically important residues are shown in red and labeled *. Residues presumed to be important for substrate binding were identified based on the crystal structure of human legumain bound to cystatin (PDB ID: 4N6O) and are highlighted in purple. The *O. affinis* AEP1$_b$ sequence is as for AEP1 (c.5.2) but with a Glu to Val substitution at position 391, as indicated by ↓. When numbering is based on wt *Oa*AEP1$_b$, this corresponds to position 371 (see Fig. 2 A).

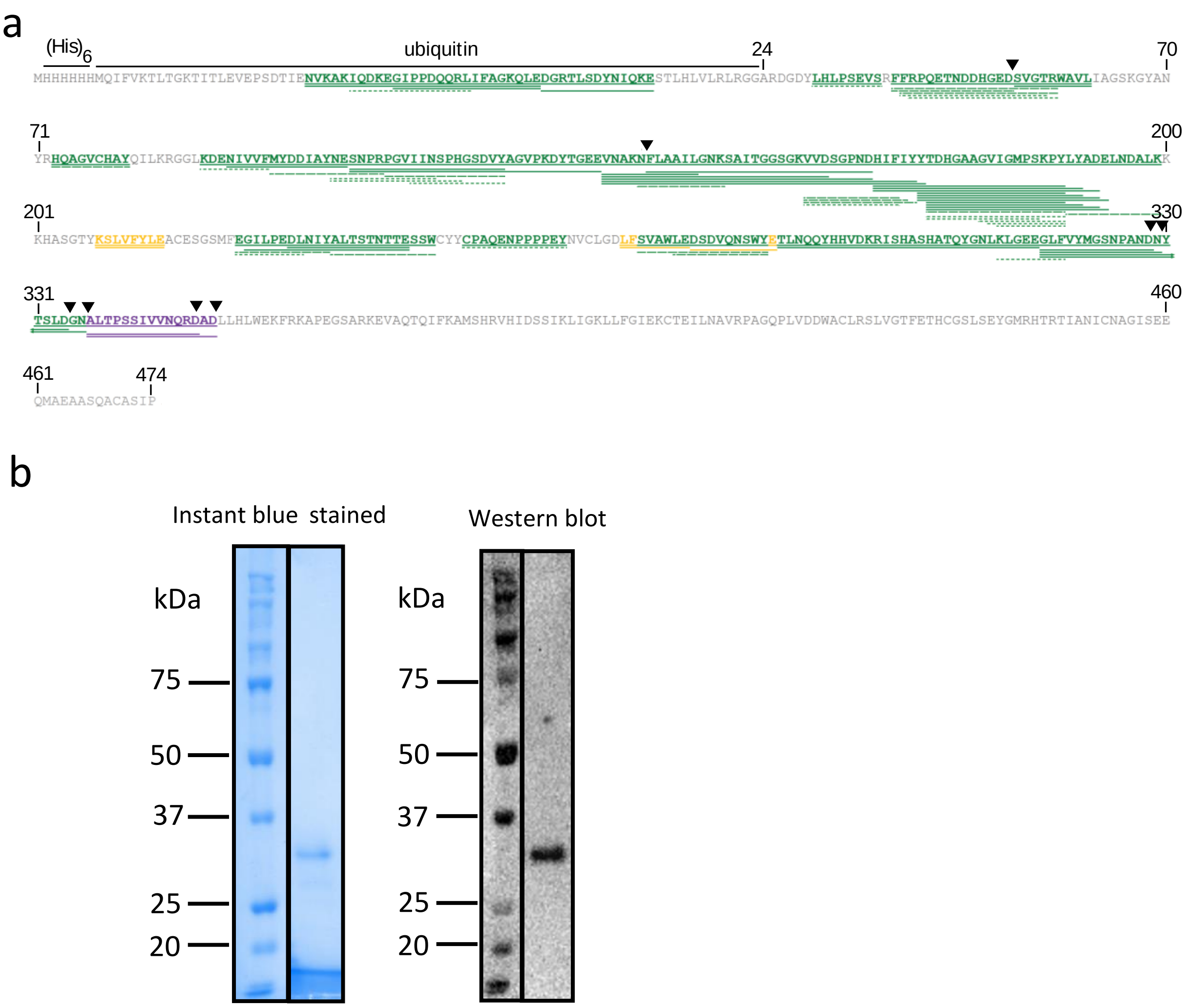


**Supplementary Figure 2:** *Expression and identification of r*Oa*AEP1$_b$.* (a) The recombinant His6-ubiquitin-*Oa*AEP1$_b$ fusion protein expressed in *E. coli* and sequence coverage attained via nanoLC-MS/MS following its enzymatic digestion. Bold text with underline denotes sequence coverage, where green or purple text indicates peptides assigned with $p > 95\%$ confidence in ProteinPilot, and yellow text indicates peptides assigned with $95 > p > 50$ confidence, but also manually verified. Lines beneath the original underlined text correspond to the sequence position of individual peptides that contribute to the overall sequence coverage. ——— denote peptides observed following endoglu-C digestion; --------- denote peptides observed following tryptic digestion; -------------- denote peptides observed following chymotryptic digestion; ▼ denote potential auto-processing sites. The Asn$_{140}$ site is unlikely to represent an authentic N-terminal processing site given its location. Arrowheads indicate continuous amino acid stretches for the corresponding peptides. Amino acid numbering is based on wt *Oa*AEP1$_b$ (see Fig. 2 a) excluding the His6-ubiquitin fusion protein tag. All MS data was calculated using the *O. affinis* AEP1$_b$ sequence, where position 371 is a Val residue. (b) Activated r*Oa*AEP1$_b$ purified by cation exchange was analysed by SDS-PAGE and (i) Instant blue staining or (ii) Western blotting with anti-*Oa*AEP1$_b$ (residues D$_{47}$ – P$_{474}$) polyclonal rabbit serum. The gel and blot are the same as those in Fig. 2 c, displayed here in their entirety.

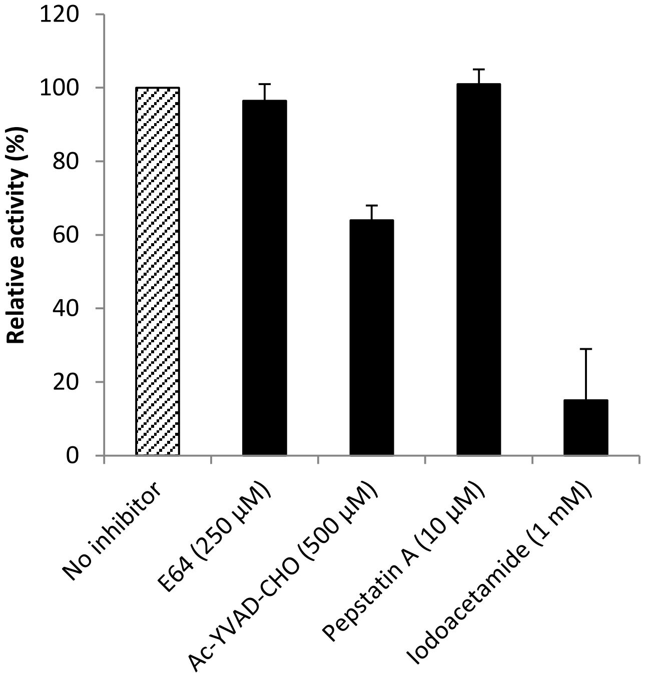


**Supplementary Figure 3:** *r*Oa*AEP1$_b$ activity against the wt IQF peptide in the presence of protease inhibitors.* r*Oa*AEP1$_b$ (~4.4 µg ml$^{-1}$ total protein) was allowed to cleave the wt IQF peptide (11 µM) and fluorescence intensity was recorded every minute for 90 min. The enzyme activity in the presence of inhibitors is reported relative to a no inhibitor control at the 90 min time point. Each data point represents the average of two technical replicates and error bars report the range.

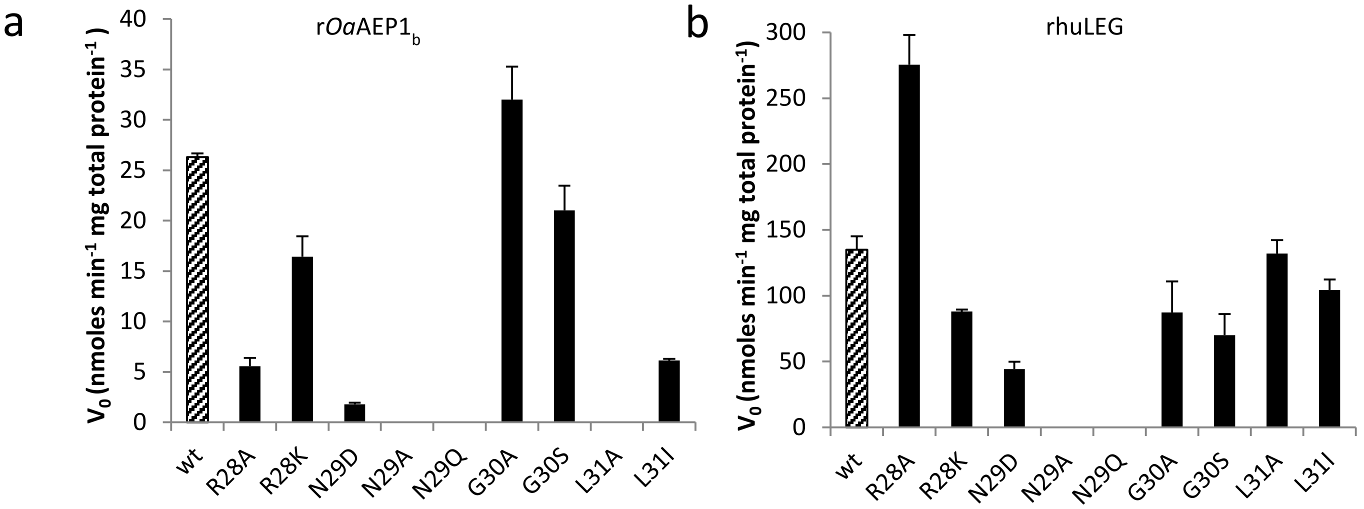


**Supplementary Figure 4:** *Comparison of substrate specificity of plant and human AEPs*. Initial velocity of (a) r*Oa*AEP1$_b$ (~3.5 µg mL$^{-1}$ total protein) and (b) rhuLEG (1.1 µg mL$^{-1}$ total protein) against 50 µM FRET peptide substrates. The average of two technical replicates are shown and the error bars report the range.

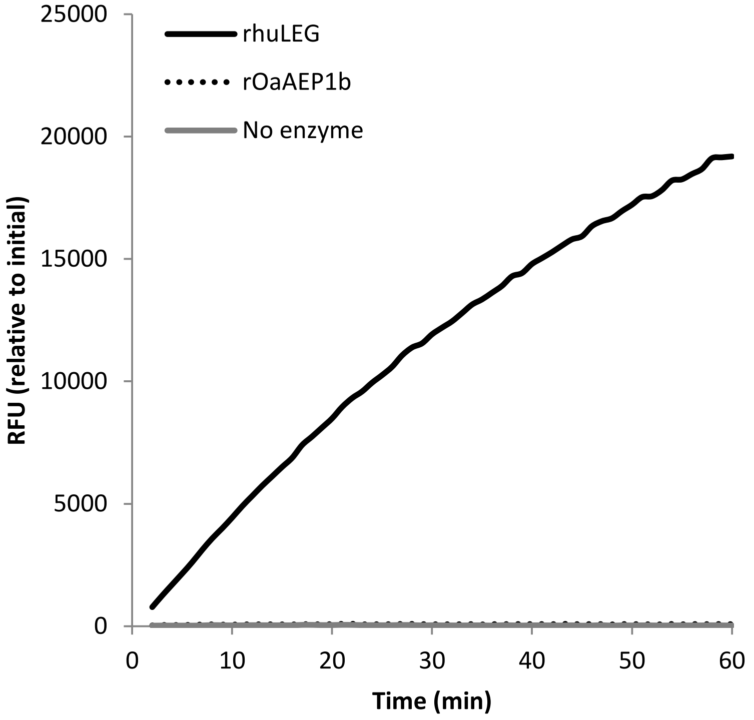


**Supplementary Figure 5:** *r*Oa*AEP1$_b$ does not cleave a substrate lacking P' residues*. Activity of r*Oa*AEP1$_b$ (~5 µg mL$^{-1}$ total protein) and rhuLEG (1.1 µg mL$^{-1}$ total protein) against the fluorogenic substrate Z-AAN-MCA (100 µM). A single representative experiment of two technical replicates is shown. RFU, relative fluorescence units.

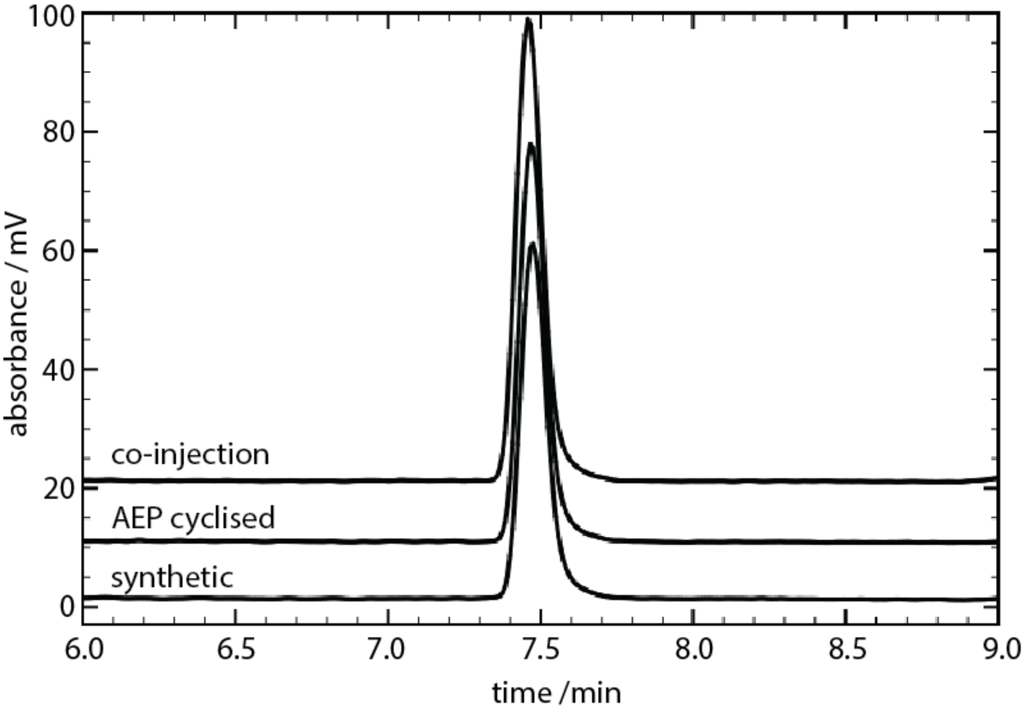


**Supplementary Figure 6:** HPLC analysis of kB1 produced *in vitro* by recombinant *O. affinis* AEP ("AEP cyclised"), synthetic kB1 and co-injection of both.

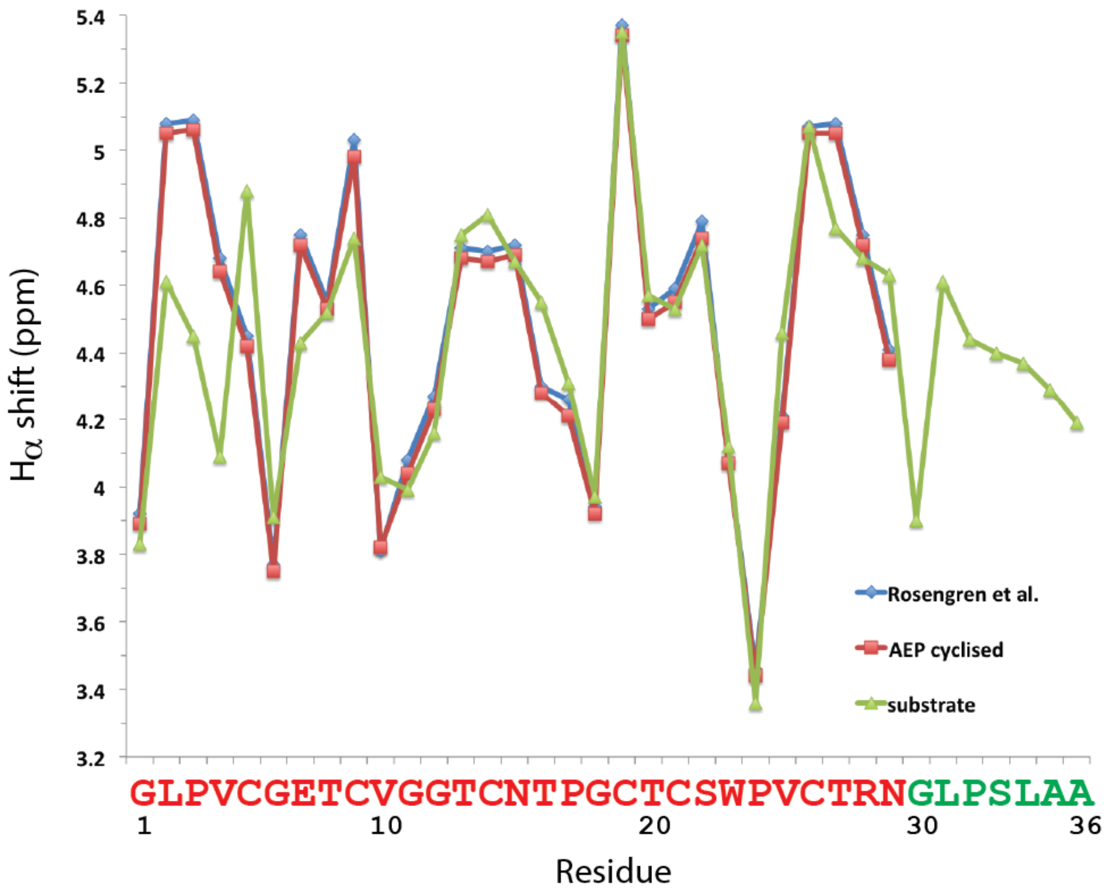


**Supplementary Figure 7 (a):** $H_a$- chemical shift comparison of kB1 produced *in vitro* by recombinant *O. affinis* AEP ( AEP cyclised), kB1 isolated from *O. affinis* ( Rosengren et al.)[1] and correctly folded linear $kB1_{wt}$ ( substrate). Minor shift differences of 0.03 ppm can be attributed to differences in pH and chemical shift referencing.

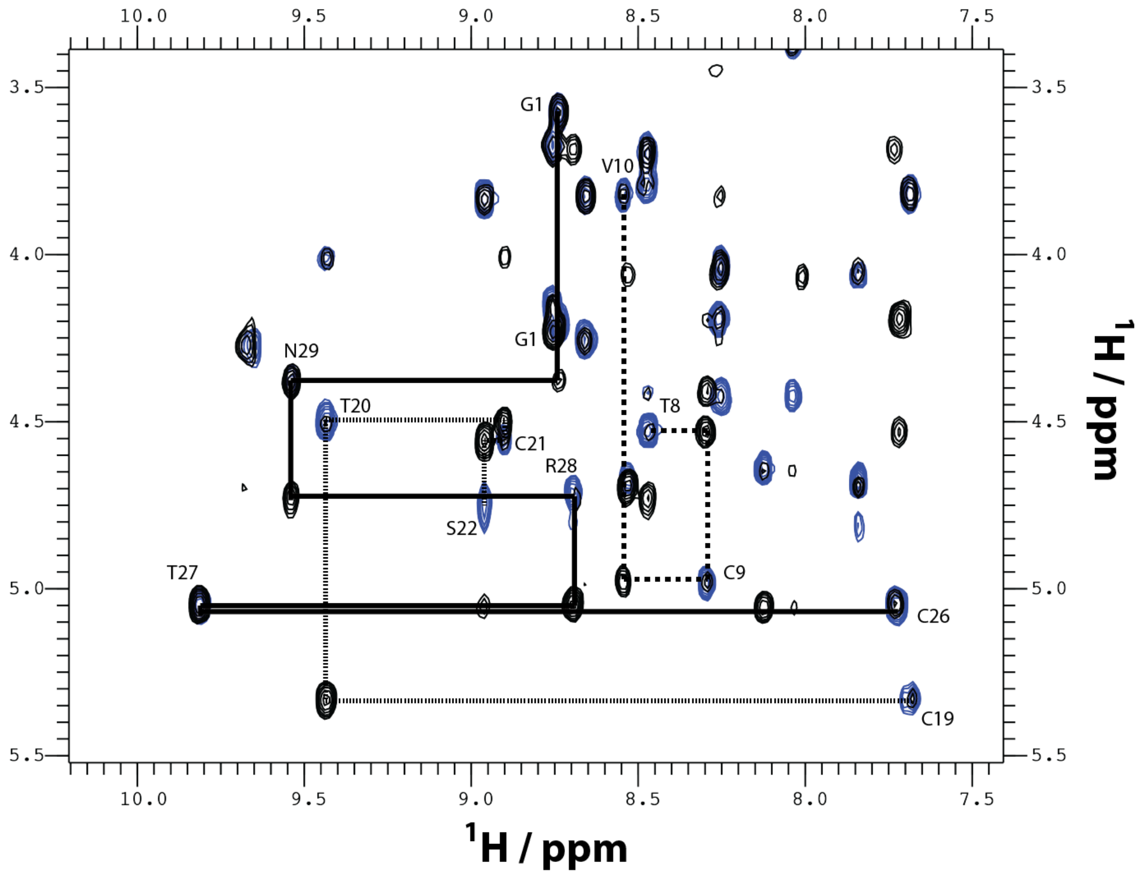


**Supplementary Figure 7 (b):** TOCSY (blue) and NOESY (black) spectra of the fingerprint region of kB1 produced *in vitro* by recombinant AEP. Representative NH-$H_\alpha$ correlations of some spin systems are labelled. The presence of a cyclic backbone is supported by the appearance of a (sequential) Asn29$^{H\alpha}$ - Gly1$^{NH}$ correlation and an Asn29$^{NH}$ - Gly1$^{NH}$ correlation (not shown).

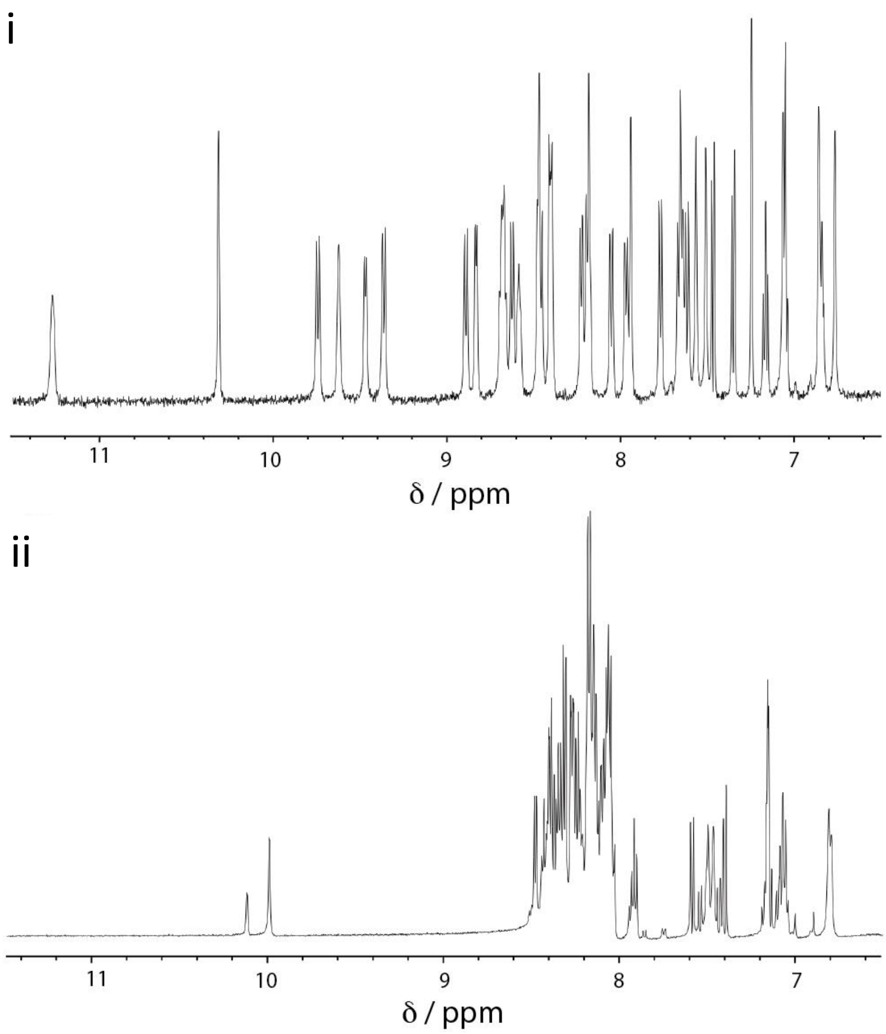


**Supplementary Figure 7 (c):** 1D-$^{1}$H NMR spectra of the aromatic/amide fingerprint region of kB1 *in vitro* cyclised by recombinant AEP (i) and kB1(6xSer)-CTR (ii).

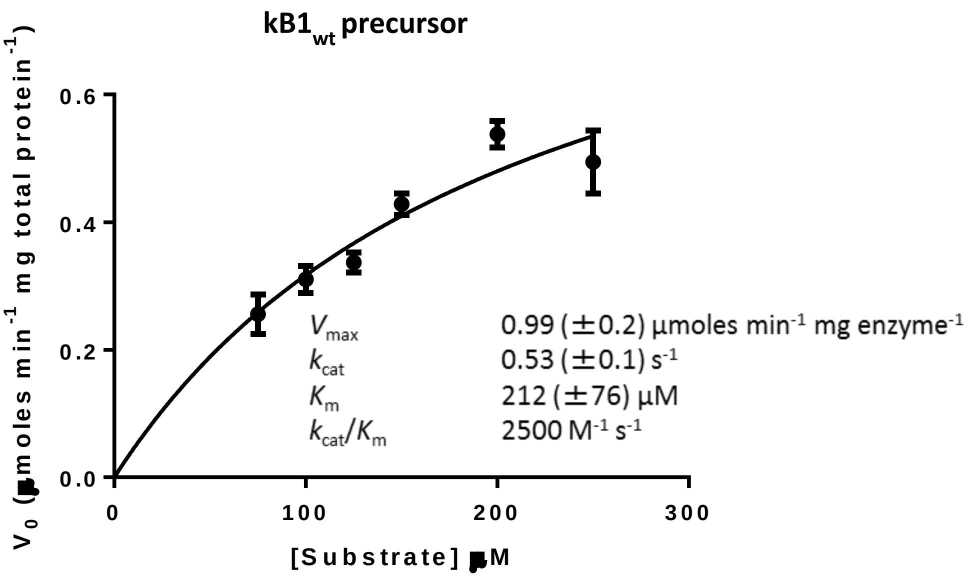


**Supplementary Figure 8:** *Kinetics of r*Oa*AEP1$_b$-mediated cyclisation.* Varying concentrations of substrate (kB1$_{wt}$ precursor) were incubated with enzyme (19.7 µg mL$^{-1}$ total protein) for 5 min. The amount of product formed was inferred by monitoring depletion of the precursor by RP-HPLC. A Michaelis-Menten plot shows the mean of three technical replicates and error bars report the SEM. The kinetic parameters derived from this plot are listed (±SEM).

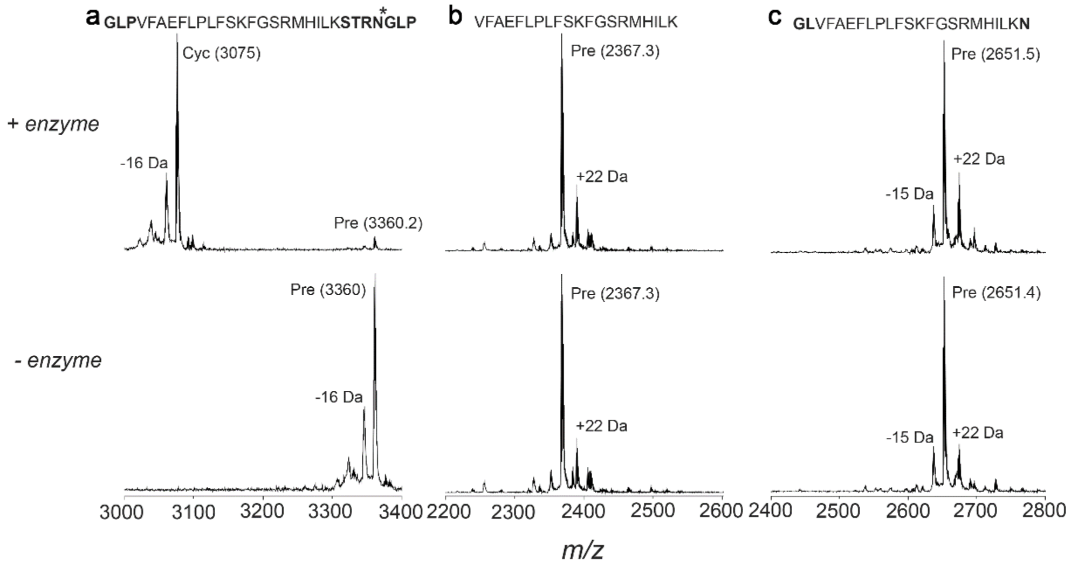


**Supplementary Figure 9:** *r*Oa*AEP1$_b$ does not cyclise a model peptide without appropriate flanking sequences.* (a-c) MALDI MS spectra of the native R1 peptide with various flanking sequences 22 h post-addition of r*Oa*AEP1$_b$ (24 µg mL$^{-1}$ total protein). Bold residues, flanking sequences. Asterisk, r*Oa*AEP1$_b$ cleavage site. Observed monoiosotopic masses (Da; $[M+H]^+$) are listed. +22 Da and -15/-16 Da peaks present in some precursor and product spectra are likely to represent $Na^+$ adducts and a synthesis-derived modification respectively. Cyc, cyclic product; Pre, linear precursor. A representative experiment of three technical replicates is shown.

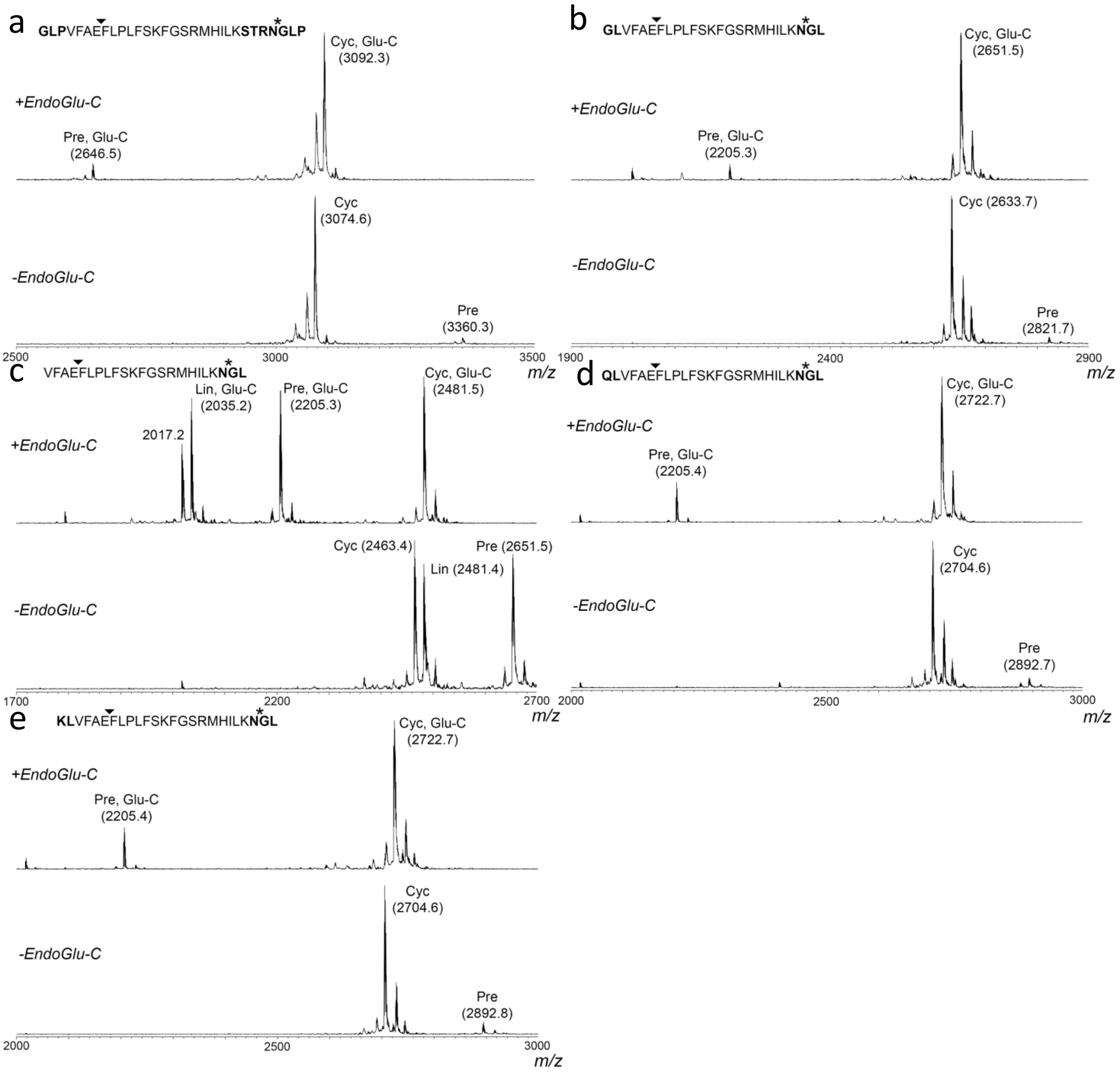


**Supplementary Figure 10:** *EndoGlu-C digestion confirms that model peptides processed by r*Oa*AEP1$_b$ are predominantly cyclic.* The R1 peptide (VFAEFLPLFSKFGSRMHILK) with various flanking sequences was incubated with r*Oa*AEP1$_b$ (24 µg mL$^{-1}$ total protein) for 22 h. Processed peptides were then incubated a further 18 h at 37$^o$C with or without the addition of EndoGlu-C and analysed by MALDI MS (a-e). Bold residues, flanking sequences. *****, r*Oa*AEP1$_b$ cleavage site. ▼, EndoGlu-C cleavage site. Observed monoiosotopic masses (Da; [M+H]$^+$) are listed for dominant peaks. Cyc, cyclic product; Pre, linear precursor; Lin, linear product; Cyc Glu-C, cyclic product hydrolysed by endoGlu-C; Pre Glu-C, linear precursor hydrolysed by endoGlu-C; Lin endoGlu-C, linear product hydrolysed by endoGlu-C. A representative experiment of two technical replicates is shown.
